# MicroRNA-221 Mediates the Effects of PDGF-BB on Migration, Proliferation, and the Epithelial-Mesenchymal Transition in Pancreatic Cancer Cells

**DOI:** 10.1371/journal.pone.0071309

**Published:** 2013-08-13

**Authors:** Anping Su, Sirong He, Bole Tian, Weiming Hu, Zhaoda Zhang

**Affiliations:** 1 Department of Hepatobiliopancreatic Surgery, West China Hospital, Sichuan University, Chengdu, P. R. China; 2 Regenerative Medicine Research Center, West China Hospital, Sichaun University, Chengdu, P. R. China; Sapporo Medical University, Japan

## Abstract

The platelet-derived growth factor (PDGF) signaling pathway has been found to play important roles in the development and progression of human cancers by regulating the processes of cell proliferation, apoptosis, migration, invasion, metastasis, and the acquisition of the epithelial-mesenchymal transition (EMT) phenotype. Moreover, PDGF signaling has also been found to alter the expression profile of miRNAs, leading to the reversal of EMT phenotype. Although the role of miRNAs in cancer has been documented, there are very few studies documenting the cellular consequences of targeted re-expression of specific miRNAs. Therefore, we investigated whether the treatment of human pancreatic cancer cells with PDGF could alter the expression profile of miRNAs, and we also assessed the cellular consequences. Our study demonstrates that miR-221 is essential for the PDGF-mediated EMT phenotype, migration, and growth of pancreatic cancer cells. Down-regulation of TRPS1 by miR-221 is critical for PDGF-mediated acquisition of the EMT phenotype. Additionally, the PDGF-dependent increase in cell proliferation appears to be mediated by inhibition of a specific target of miR-221 and down-regulation of p27Kip1.

## Introduction

Pancreatic cancer is the fourth most common cause of cancer-related death in the United States [1], and the aggressiveness of pancreatic cancer is in part due to its intrinsic and extrinsic drug resistance characteristics, which are also associated with the acquisition of the epithelial-to-mesenchymal transition (EMT) phenotype [2], [3]. The EMT is a process that is suggestive of cancer cells’ stem-like characteristics, whereby epithelial cells with a cobblestone phenotype acquire mesenchymal cell characteristics with a spindle-shaped fibroblast-like morphology [3], [4]. This process involves the disassembly of cell-cell junctions, including the down-regulation of epithelial cell phenotype markers (E-cadherin, zonula occludens-1), as well as the translocation of β-catenin from the cellular membrane to the nucleus, reorganization of the actin cytoskeleton, and up-regulation of mesenchymal cell phenotype markers (vimentin, fibronectin and N-cadherin) [4]. This process reduces the adhesive capacity of mesenchymal phenotypic cells, which leads to increased cell migration and invasion, resulting in tumor aggressiveness [5].

Platelet-derived growth factor (PDGF) can regulate many cellular processes, including cell proliferation, transformation, migration, invasion, angiogenesis and metastasis, by activating its cognate receptor [6]. In recent years, PDGF-BB and the cognate tyrosine kinase β-receptors have been found to play important roles in the acquisition of the epithelial-mesenchymal transition (EMT) phenotype of cancer cells [5], [7]. The treatment of epithelial-like cancer cells with purified PDGF protein resulted in significantly decreased expression of E-cadherin and increased expression of the zinc-finger E-box binding homeo-box 2 (ZEB2) at both the mRNA and protein levels in association with the induction of EMT characteristics [8]. ZEB2 has been shown to be involved in the down-regulation of the expression of many genes coding for crucial proteins of the epithelial cell phenotype, including E-cadherin, concomitant with up-regulation of the expression of vimentin [9]. It is important to note that PDGF also significantly increased the expression of fibronectin, N-cadherin, and vimentin, with concomitant loss of E-cadherin. However, further in-depth mechanistic studies are required for understanding how PDGF regulates the processes involved in the EMT phenotype.

MicroRNAs (miRNAs) have been identified that enhance several aspects of pancreatic cancer pathogenesis, including metastasis, invasion, and self-renewal [10]. Emerging evidence suggests that the expression of genes that are fundamental to the acquisition of the EMT phenotype and tumor cell aggressiveness are regulated by miRNAs that lead to either translational repression or the degradation of target mRNAs [11], [12]. The miR-221 is highly expressed in various cancer-derived cells, including pancreatic carcinoma, prostate carcinoma and thyroid carcinoma cells [13], [14], [15]. Recent studies have shown that miR-221 regulates the EMT by targeting TRPS1 (tricho-rhino-phalangeal syndrome type 1) and relieving its inhibition of ZEB2 [16]. Moreover, high levels of miR-221 have been shown to promote proliferation of these cells through binding to the 3′-UTR of the cell cycle inhibitor and tumor suppressor p27Kip1 and inhibiting its expression [13], [15]. In recent years, PDGF signaling has been found to alter the expression profile of miRNAs, leading to the reversal of the EMT phenotype [17], [18].

Although the role of miRNAs in cancer has been documented, there are very few studies documenting the cellular consequence of targeted re-expression of specific miRNAs. Therefore, we hypothesized that PDGF signaling might modulate the EMT phenotype via regulation of miRNA biogenesis. In this study, we investigated whether the treatment of human pancreatic cancer cells with PDGF could alter the expression profile of miRNAs, and we also assessed the cellular consequences. The results demonstrate that PDGF exhibits its effects on both cell proliferation and the EMT phenotype by inducing miR-221 expression, which results in down-regulation of p27Kip1 and TRPS1 in pancreatic cancer cells.

## Materials and Methods

### Cell Culture

The stable human pancreatic cancer AsPC-1 cell lines obtained from Typical culture preservation commission cell bank, Chinese academy of sciences (Shanghai, China) were cultured in DMEM medium (Invitrogen, CA) supplemented with 5% fetal bovine serum (FBS), 2 mmol/l glutamine, 50 units/ml penicillin, and 50 µg/ml streptomycin. All of the cells were maintained in a 5% CO2-humidified atmosphere at 37°C.

### Research Reagents and Antibodies

Recombinant human PDGF-BB was purchased from R&D Systems. Chemically synthesized miRNA inhibitors, mimics, and scrambled controls were obtained from Dharmacon or Ambion. The synthetic small interference RNAs (siRNAs) targeting human TRPS1 or p27Kip1 were Stealth Select RNAi (Invitrogen) and HP Validated siRNA (Qiagen). Cells were seeded at 100,000 to 300,000 cells/ml and transfected by Oligofectamine (Invitrogen), DharmaFECT3 transfection reagent (Dharmacon) or RNAi MAX (Invitrogen) at the indicated concentrations. Antibodies against ZEB2, N-cadherin, and vimentin were purchased from Cell Signaling Technology (Beverly, MA). Antibodies against ZEB1, Snail1, Snail2, Twist, E47, E-cadherin and ZO-1 were purchased from Santa Cruz. Antibodies against TRPS1, p27Kip1 and GAPDH were acquired from Abcam. FITC-conjugated anti-mouse IgG for vimentin and E-cadherin staining was purchased from Invitrogen. The miRVANA miRNA Isolation Kit and Taqman miRNA Assays were purchased from Ambion and Applied Biosystems.

### Transfection of miRNA Mimics and Specific Anti-miRNAs

AsPC-1 cells were seeded at 3×10^5^ cells per well in six-well plates and transfected with miR-221 mimics or miRNA scrambled controls at a final concentration of 20 nM using Oligofectamine (Invitrogen). AsPC-1 cells were transfected with anti-miR221 (miRNA inhibitors) or an anti-miRNA control at a final concentration of 200 nM using the DharmaFECT3 transfection reagent (Dharmacon). After 3 days of transfection, the cells were split and transfected repeatedly with miR-221, miRNA inhibitors, or control every 3–4 days for the indicated times.

### Small Interfering RNA and Transfection

AsPC-1 cells were transfected with 100 nmol/l human TRPS1, p27Kip1 small interfering (si)RNA or a control siRNA (Santa Cruz) using RNAi MAX transfection reagent. The media were removed after a 24-hour transfection, and then the cells were incubated in media containing 5% FBS for another 24 hours. Cell lysates were prepared for Western blot analysis, and total RNA was extracted for the real-time reverse transcription-polymerase chain reaction assay.

### Cell Proliferation Assay

The MTT assay was performed as described previously [19]. In total, 6×10^3^ cells were split into 96-well plates. The cells were treated with or without 20 ng/ml PDGF-BB for 24 h, followed by a cell proliferation assay using a CellTiter96 nonradioactive cell proliferation assay (Promega) according to the manufacturer’s directions. The absorbance at 490 nM was read by an enzyme-linked immunosorbent assay plate reader. For the proliferating cell nuclear antigen (PCNA) staining [20], AsPC-1 cells were stained with a FITC-conjugated anti-PCNA antibody (clone PC10) from Biolegend as well as DAPI. At least 150 cells were counted per condition, and the percentages of PCNA-positive cells are presented.

### Wound Healing Assay

A wound healing assay was performed to examine the capacity of cell migration and invasion, as described previously [21]. Briefly, after the cells grew to 90–95% confluence in 6-well plates, a single scratch wound was generated with a 200-µl disposable pipette tip. The scratch wounds were photographed over 7 h with a Nikon inverted microscope with an attached digital camera, and their widths were quantitated with the ImageJ software. The data were plotted as the percentage of wound closure, setting the initial scratch width as 100%. The results are presented as the mean of triplicate measurements per condition in three independent experiments.

### Cell Invasion Assay

The invasive behaviors of the cells were tested using a Matrigel transmembrane invasion assay. Transwell chambers (Millipore) (8 µm pore size) were coated with Matrigel (15 µg/filter). Cells (2.0×10^4^) in serum-free medium were plated into the upper chamber and bottom wells were filled with complete medium. Cells were allowed to invade across the Matrigel-coated membrane for 72 h at 37°C in 5% CO2. After incubation, cells were removed from the upper surface of the filter by scraping with a cotton swab. The invaded cells that adhered to the bottom of the membrane were fixed with methanol and stained with DAPI. The number of cells that penetrated the membrane was determined by counting the mean cell number of five randomly selected high-power fields.

### Quantitative Real-time PCR

RNA was purified using RNeasy (Qiagen) or miRVANA (Ambion) kits with DNase digestion on RNeasy columns. Complementary DNA (cDNA) was generated with the High-Capacity cDNA Reverse Transcription kit (Roche). The quantitative analysis of the change in expression levels was calculated by the real-time PCR machine (iQ5, Bio-Rad). The PCR cycling conditions were 94°C for 3 min and 40 cycles of (94°C for 15 s, 60°C for 20 s and 72°C for 40 s). Real-time PCR was used to quantify the mRNA expression. The reactions were performed in duplicate, and the delta-delta-Cycle Threshold (ddCt) values were calculated on the basis of the average of the normalization genes. Taqman miRNA assays were used for the quantitation of miR-221, and the results were normalized to the average of the results obtained for RNU6B.

### Western Blot Analysis

Western blot analysis was performed using total cell lysates. Total cell lysates from different experiments were obtained by lysing the cells in RIPA buffer containing 50 mM Tris–HCl, 150 mM NaCl, 1% NP-40, 0.1% SDS, 0.5% sodium deoxycholate, 2 mM sodium fluoride, 2 mM Na3VO42, 1 mM EDTA, 1 mM EGTA, and 1 mM protease inhibitor cocktail. The total cell lysates were separated on SDS-PAGE gels, transferred to PVDF membranes (Millipore), immunoblotted with antibodies, and visualized using an enhanced chemiluminescence detection system (Amersham Biosciences). The protein bands were quantitated by densitometry using gel analysis software ImageJ (rsbweb.nih.gov/ij). All values were normalized to GAPDH.

### Immunofluorescence Microscopy

Briefly, the cells were fixed with 4% paraformaldehyde, permeabilized in 0.5% Triton X-100 and then blocked with 10% goat serum. The cells were incubated for 1 hour with antibodies against E-cadherin (1∶200) or vimentin (pre-diluted) in 5% goat serum, and then they were stained for 1 hour with a FITC-conjugated secondary antibody (1∶250). The cells were viewed by fluorescence microscopy, and the images were analyzed using Advanced Sport software (Diagnostic Instruments, Sterling Heights, MI).

### Statistical Analysis

The results presented are the average of at least three experiments, each performed in triplicate with standard errors. Statistical analyses were performed by analysis of variance followed by Tukey’s multiple comparison test or Student’s t test using SPSS15.0. p values of 0.05 were considered significant and are indicated with asterisks.

## Results

### PDGF Promoted Cell Migration and Proliferation and Altered the Epithelial- Mesenchymal Transition Phenotype in AsPC-1 Cells

**Figure 1:**
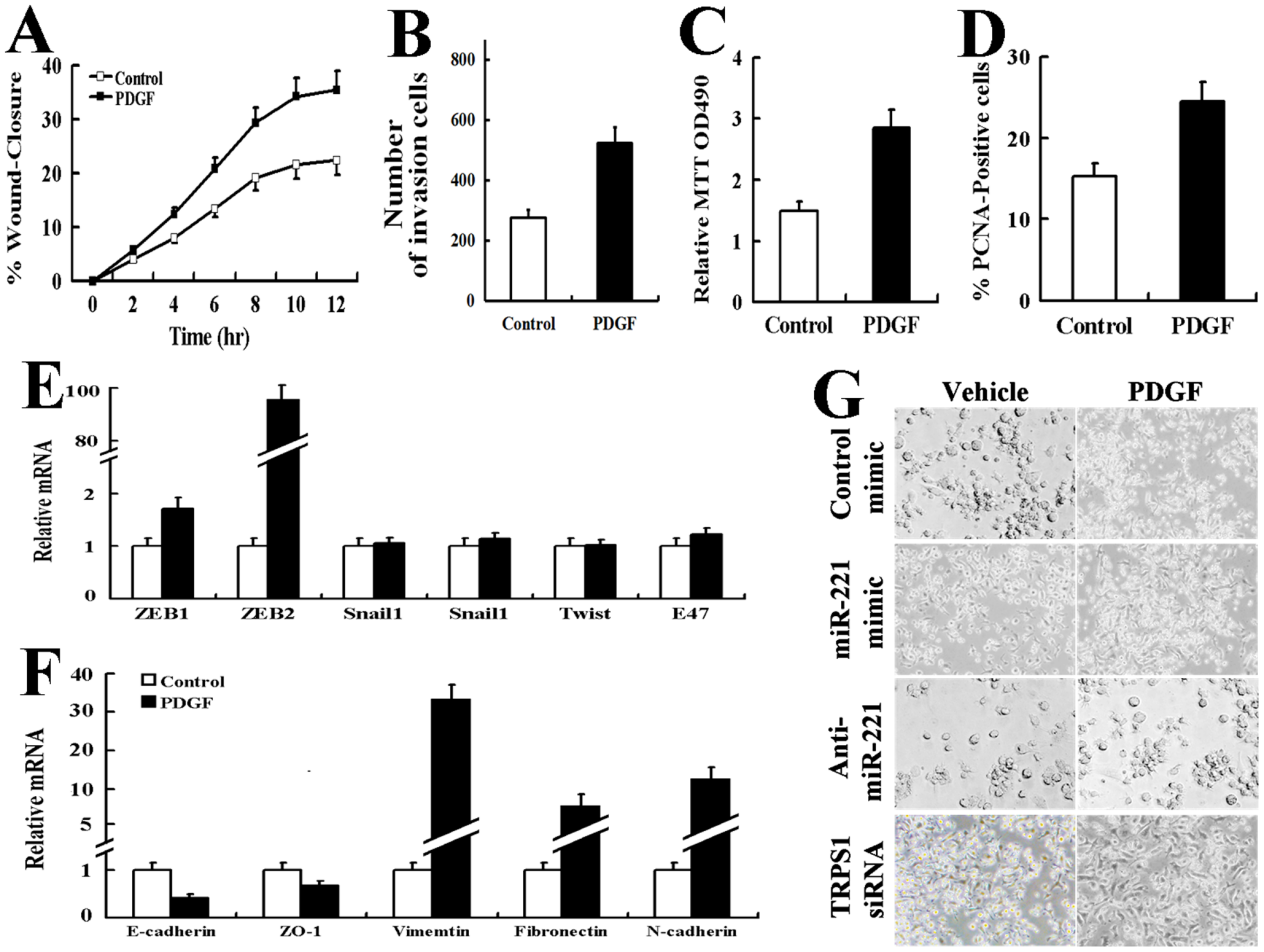
PDGF promoted cell migration and proliferation and altered the EMT phenotype in AsPC-1 cells. AsPC-1 cells incubated with vehicle or PDGF-BB (20 ng/ml) were subjected to the scratch wound assay (A) and Matrigel transmembrane invasion assay (B). The results are the mean±SD. of triplicate measurements of three independent experiments. AsPC-1 cells incubated with vehicle or PDGF-BB (20 ng/ml) for 24 h were subjected to the MTT cell proliferation assay (The results are indicated as the absorbance readings at 490 nm) (C) or stained with a FITC-conjugated antibody against the proliferation marker PCNA and DAPI (150 cells were counted per condition, and the percentage of PCNA-positive cells is presented.) (D). The results are the mean±SD. for triplicate assays of three independent experiments. Real time-PCR was used to determine the mRNA levels to evaluate the upregulated expression of transcription factors (E) and EMT-specific genes (F) in AsPC-1 cells incubated with vehicle or PDGF-BB (20 ng/ml, 24 hr). The relative mRNA levels were normalized to GAPDH. All of the treatments in this figure were carried out in triplicate, and the results are displayed as the means ± SD. G: Photomicrographs of AsPC-1 cells incubated with vehicle or PDGF-BB (20 ng/ml, 24 hr). Original magnification, 200X.

We first examined whether PDGF promotes cell migration using an in vitro scratch wound assay and Matrigel transmembrane invasion assay. In AsPC-1 cells, treatment with PDGF strongly promoted cell migration (Fig. 1A, B). We also determined whether cell proliferation was enhanced by PDGF-BB (20 ng/ml, 24 hr) in AsPC-1 cells. The results of the MTT cell proliferation assay indicated that PDGF increased the number of viable cells 1.9-fold compared with the control (Fig. 1C). We then confirmed these results using quantitative analysis of PCNA staining. AsPC-1 cells treated with vehicle or PDGF-BB (20 ng/ml) for 24 h were stained with PCNA to measure the number of proliferating cells (Fig. 1D). The result showed the PDGF-mediated increase in cell proliferation (1.6-fold) was consistent with the results of the MTT assay in Fig. 1B.

In this study, using real-time PCR, we also found that PDGF-BB (20 ng/ml, 24 hr) treatment significantly upregulated the expression of transcriptional repressors in the processes of the EMT, inducing genes such as ZEB2 in AsPC-1 cells (Fig. 1E). We found that it has no change in the expression of Snail 1, Snail 2, Twist and E47. These changes in expression were concomitant with the loss of E-cadherin and zonula occludens-1 and with the gain of vimentin, fibronectin and N-cadherin expression (Fig. 1F). Most importantly, AsPC-1 cells treated with PDGF-BB displayed an elongated/irregular fibroblastoid morphology, which showed an epithelial cobblestone appearance (Fig. 1G). These results suggest that PDGF led to the acquisition of the EMT phenotype.

### Regulation of miRNA Expression by PDGF-BB Treatment in AsPC-1 Cells

**Figure 2:**
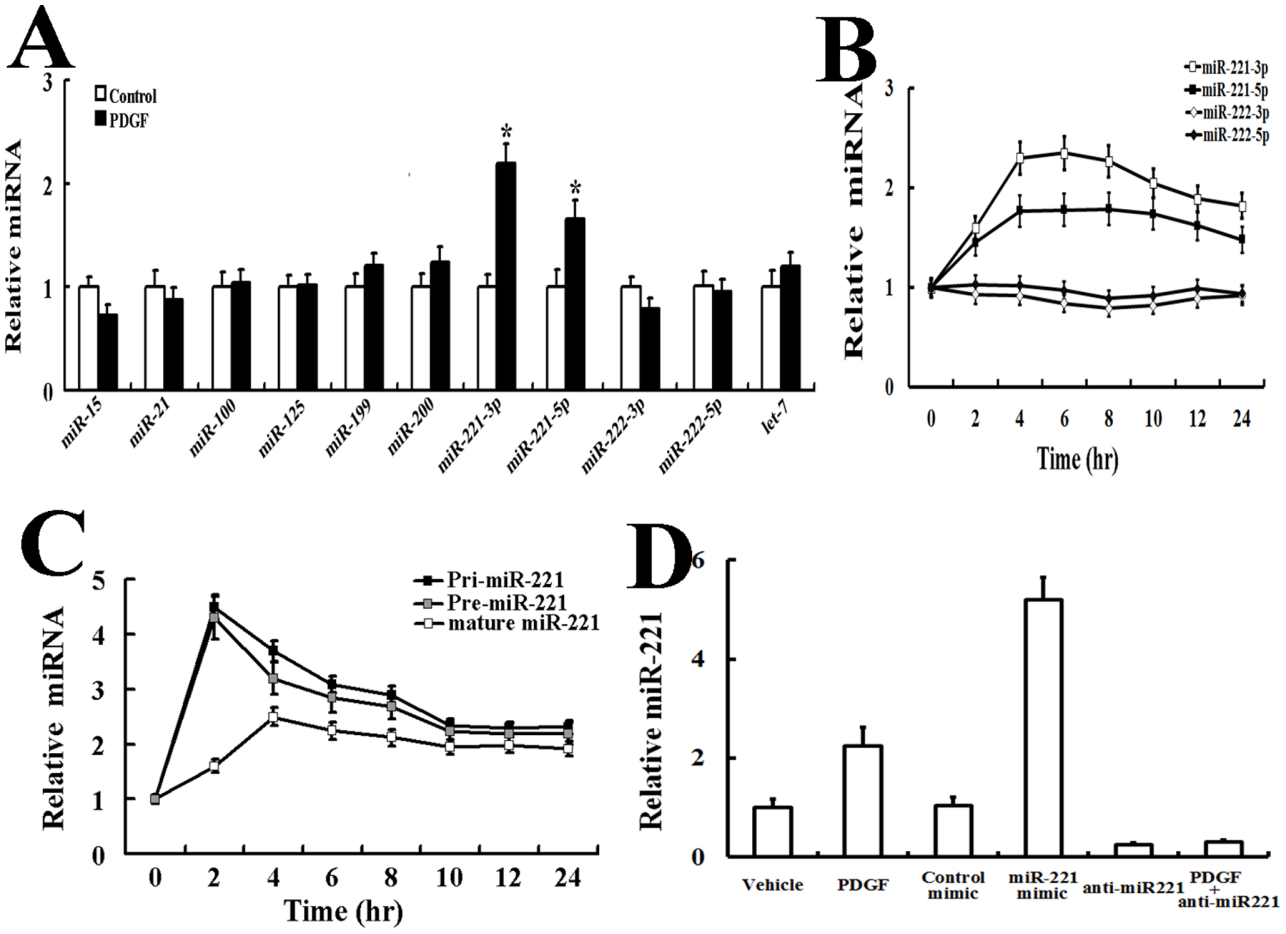
miR-221 is regulated by the PDGF-BB signaling pathway. A: The relative miRNA levels in AsPC-1 cells treated with vehicle or PDGF-BB (20 ng/ml, 24 hr) B, C: Time-course expression of the relative expression of miR-221-3p, miR-221-5p, miR-222-3p and miR-222-5p (B), and miR-221 transcripts (Pri-miR-221), Pre-miR-221 or mature miR-221 (C) normalized to GAPDH (for Pri-miR-221 or Pre-miR-221), or U6 small nuclear RNA (for miR-221-3p, miR-221-5p, miR-222-3p, miR-222-5p and mature miR-221) in AsPC-1 cells treated with vehicle or PDGF-BB (20 ng/ml, 24 hr). D: AsPC-1 cells were treated with vehicle or PDGF-BB (20 ng/ml), or transfected with a negative control, the miR-221 mimic, anti-mIR-221, or anti-mIR-221 and followed by PDGF-BB treatment., and subjected to qRT-PCR of miR221. All of the treatments in this figure were carried out in triplicate, and the results are displayed as the means ± SD.

Real-time PCR was used to determine whether treatment of cells with PDGF-BB (20 ng/ml, 24 hr) could alter the expression of miRNA compared with untreated cells. We found that miR-221-3p and miR-221-5p were two in the few miRNAs enriched in AsPC-1 cells treated with PDGF-BB (Fig. 2A), suggesting that miR-221 expression might be activated by PDGF signaling. A time-course of the expression of miR-221-3p, miR-221-5p, miR-222-3p and miR-222-5p was examined after PDGF-BB treatment in AsPC-1 cells. MiR-221-3p and miR-221-5p were induced 2.4-fold and 1.7-fold respectively after 4 h of treatment with PDGF and gradually decreased by 10 h (Fig. 2B). Importantly, robust induction of both the primary transcripts (Pri-miR-221) and the intermediate product (Pre-miR-221) of miR-221 was observed as early as 2 h after PDGF treatment, suggesting that miR-221 is likely to be transcriptionally induced by PDGF signaling (Fig. 2C).

### miR-221 is Essential for the PDGF-mediated Epithelial-mesenchymal Transition Phenotype, Migration, and Growth of AsPC-1 Cells

**Figure 3:**
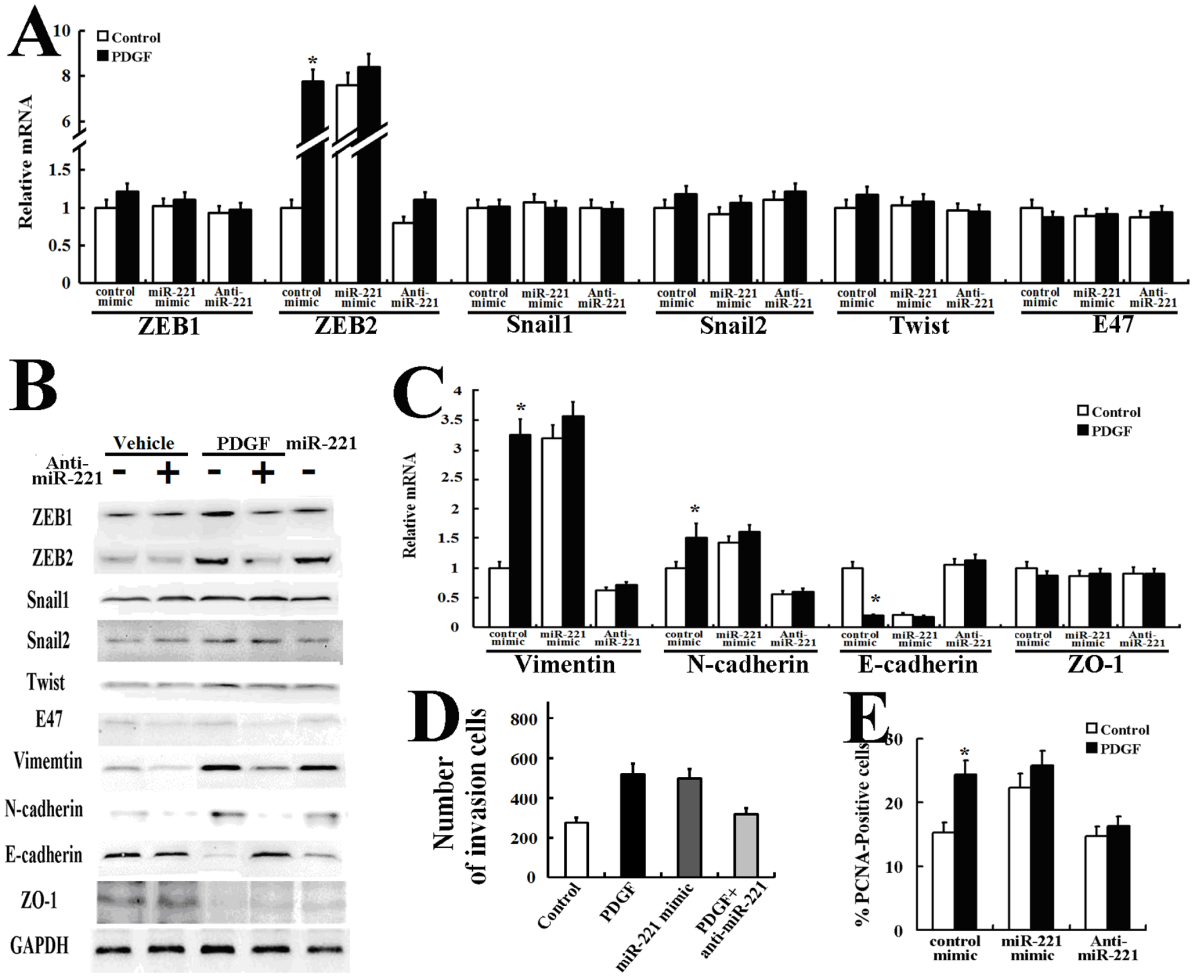
miR-221 is critical for the PDGF-mediated epithelial-mesenchymal transition phenotype, migration, and growth of AsPC-1 cells. AsPC-1 cells were transfected with a negative control, the miR-221 mimic, or antisense oligonucleotides to miR-221 (anti-mIR-221). The cells were then treated with PDGF-BB (20 ng/ml) for 24 h and subjected to qRT-PCR (A, C) or Western blot analysis (B) of the transcription factors and EMT-specific gene markers. AsPC-1 cells were transfected with a negative control, the miR-221 mimic, or antisense oligonucleotides to miR-221 (anti-mIR-221) and subjected to the Matrigel transmembrane invasion assay (D) in the presence or absence of 20 ng/ml PDGF-BB. AsPC-1 cells were transfected with a negative control, the miR-221 mimic, or antisense oligonucleotides to miR-221 (anti-mIR-221), followed by PDGF-BB treatment for 24 h, and then were stained with a FITC-conjugated antibody against the proliferation marker PCNA and DAPI. In total, 150 cells were counted per condition, and the percentage of PCNA-positive cells (E) is presented. All treatment experiments in this figure were carried out in triplicate, and the results are displayed as the means ± SD.

We transfected AsPC-1 cells with synthetic miR-221 (miR-221 mimic) or control mimic (containing a sequence from GFP) and examined the EMT phenotype. Exogenous miR-221 upregulated the expression of ZEB2 and vimentin mRNA and protein, and it reduced the expression of E-cadherin mRNA and protein, similar to the effect of PDGF (Fig. 3A, B, C). Next, RNA oligonucleotides against miR-221 (anti-miR-221) were transfected into AsPC-1 cells to inhibit miR-221 function. Anti-miR-221 transfection reduced both the basal and PDGF-induced expression of miR-221 by more than 70% (Fig. 2C). Western blot analysis (Fig. 3B) and real-time PCR (Fig. 3C) indicated that the PDGF-BB-mediated EMT phenotype was significantly impaired when miR-221 was inhibited. These results confirm that miR-221 plays an essential role in the PDGF-mediated EMT phenotype.

PDGF not only can mediate the EMT phenotype but also can promote cell migration and proliferation. Therefore, AsPC-1 cells were transfected with a miR-221 mimic, anti-miR-221, or anti-GFP (control) to examine whether induction of miR-221 by PDGF plays a role in cell migration and proliferation. When miR-221 function was blocked by anti-miR-221, PDGF did not alter the level of cell migration and proliferation, suggesting that induction of miR-221 is essential for PDGF-dependent cell migration (Fig. 3D) and proliferation (Fig. 3E). Conversely, transfection of the miR-221 mimic alone significantly elevated cell migration (Fig. 3D) and proliferation (Fig. 3E) to a level similar to that of untransfected cells treated with PDGF. These results indicate that PDGF-dependent induction of miR-221 is essential for promoting cell migration and proliferation.

Thus, miR-221 mediates the effects of PDGF on the migration, proliferation, and differentiation of AsPC-1 cells. This result raises the question of whether a single miR-221 target may be responsible for all of these phenomena or whether miR-221 might dispatch the signal through multiple functionally and physically distinct targets.

### MiR-221 Promotes the EMT by Targeting TRPS1

**Figure 4:**
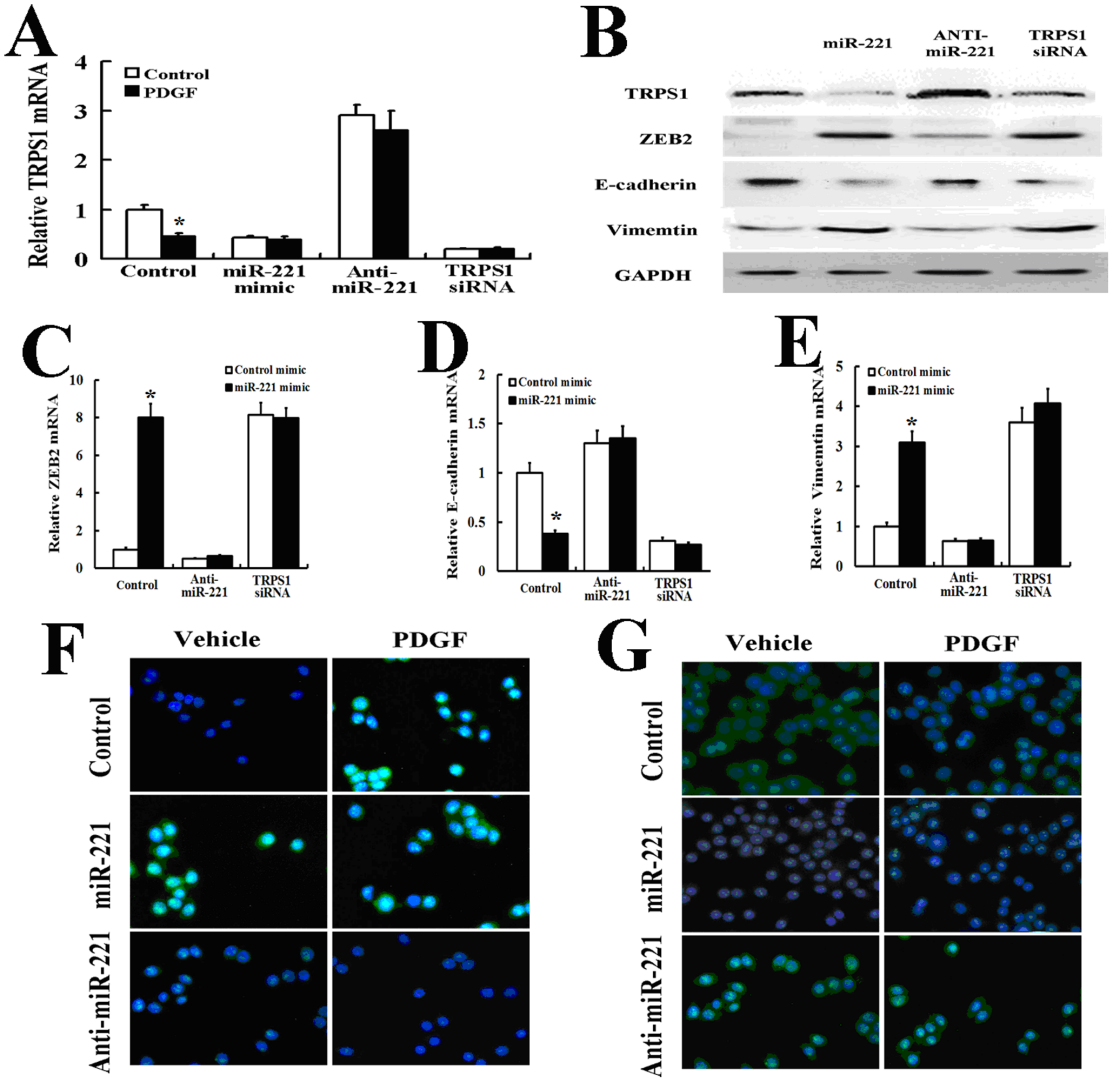
PDGF mediated the EMT by miR-221 targeting TRPS1. AsPC-1 cells were transfected with a negative control mimic, the miR-221 mimic, antisense oligonucleotides to miR-221 (anti-mIR-221) or TRPS1 siRNA. The cells were then treated with PDGF-BB (20 ng/ml) for 24 h and subjected to qRT-PCR of TRPS1. AsPC-1 cells were transfected with a negative control mimic, the miR-221 mimic, antisense oligonucleotides to miR-221 (anti-mIR-221) or TRPS1 siRNA for 3 days and subjected to Western blot analysis of TRPS1, ZEB2, E-cadherin and vimentin. AsPC-1 cells were transfected with antisense oligonucleotides to miR-221 (anti-mIR-221). The cells were then treated with a negative control or miR-221 mimic for 3 days and subjected to qRT-PCR for ZEB2 (C), E-cadherin (D) and vimentin (E). AsPC-1 cells were transfected with a negative control mimic, the miR-221 mimic or antisense oligonucleotides to miR-221 (anti-mIR-221). The cells were then treated with PDGF-BB (20 ng/ml) for 24 h and subjected to immunofluorescence staining for vimentin (F) and E-cadherin (G) All treatments in this figure were carried out in triplicate, and the results are displayed as the means ± SD.

A previous study has shown that the GATA family transcriptional repressor TRPS1 can inhibit the EMT by decreasing ZEB2 expression [16], [22]. MiR-221 has previously been shown to target TRPS1 mRNA, leading to reduced expression of the gene products through mRNA degradation and/or translational inhibition [16]. We first investigated whether the TRPS1 mRNA or protein levels are modulated by PDGF-BB in AsPC-1 cells. The TRPS1 mRNA level was decreased by approximately 55% (Fig. 4A) upon treatment of AsPC-1 cells with PDGF-BB for 24 h under conditions that significantly inhibited the expression of EMT markers (Fig. 3B, C). Therefore, TRPS1 is regulated by PDGF signaling. To assess whether miR-221 is responsible for the PDGF-dependent down-regulation of TRPS1, the miR-221 mimic was transfected into AsPC-1 cells, and then the protein and mRNA levels of TRPS1, ZEB2, E-cadherin and vimentin were examined by Western blot analysis and real-time PCR. Exogenous miR-221 significantly reduced the expression of TRPS1 and E-cadherin and increased the expression of ZEB2 and vimentin (Fig. 4B, C, D, E). On the contrary, when more than 70% of endogenous miR-221 was depleted by anti-miR-221, the levels of TRPS1 mRNA and protein were elevated (Fig. 4A, B), and the AsPC-1 cells presented the EMT phenotype (Fig. 4F, G). Moreover, we observed that exogenous miR-221 did not further alter the expression of the EMT phenotype after TRPS1 knockdown by siRNA. More importantly, PDGF-BB treatment did not repress TRPS1 in the presence of anti-miR-221 (Fig. 4A). These results demonstrated that miR-221 directly targets TRPS1 mRNA, as previously observed [16].

### PDGF-mediated Inhibition of p27Kip1 by miR-221 Promotes Cell Proliferation

**Figure 5:**
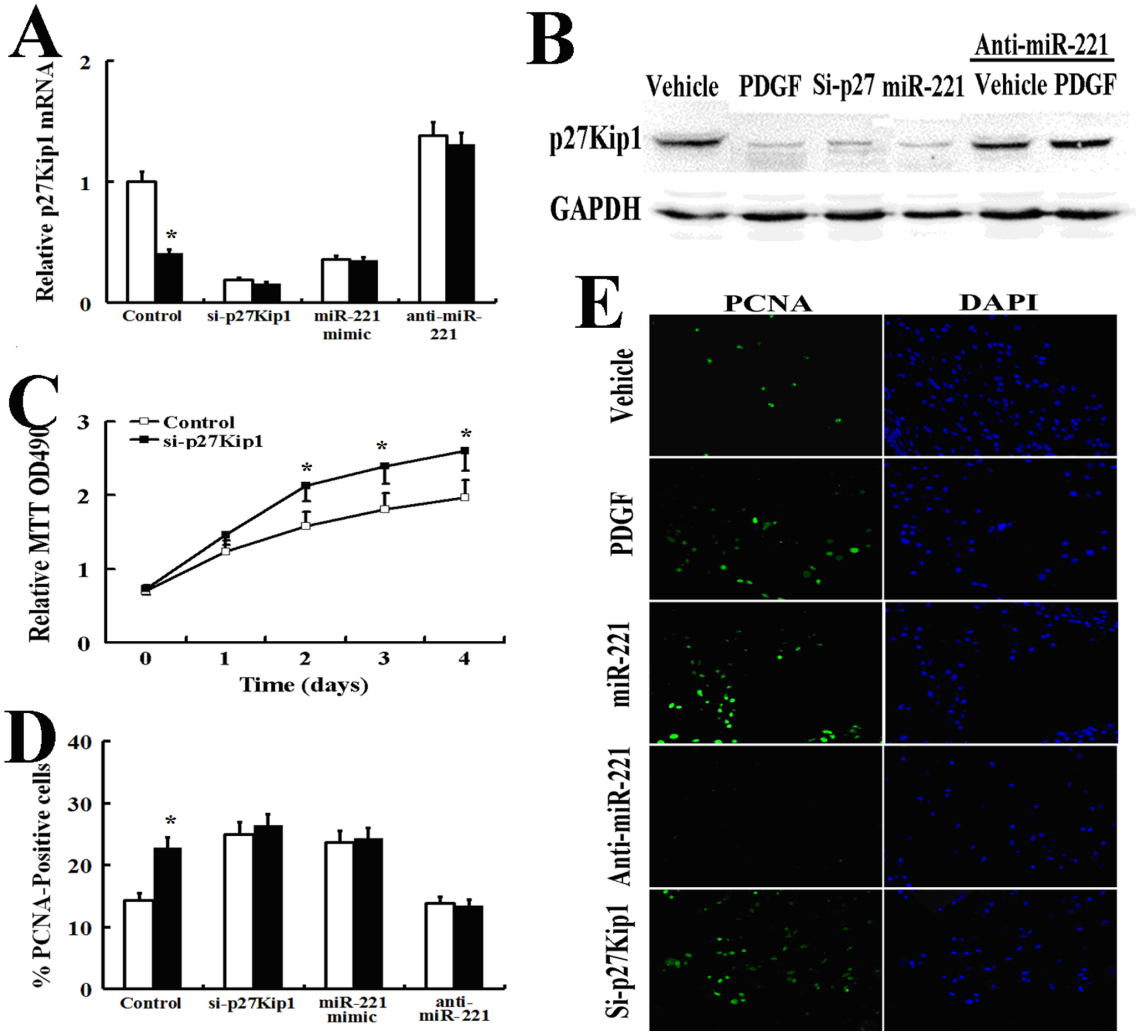
PDGF-mediated inhibition of p27Kip1 by miR-221 Promotes Cell proliferation. (A, B): AsPC-1 cells were transfected with a negative control mimic, the miR-221 mimic, antisense oligonucleotides to miR-221 (anti-mIR-221) or p27Kip1 siRNA. The cells were then treated with PDGF-BB (20 ng/ml) for 24 h and subjected to qRT-PCR (A) and Western blot analysis (B) for p27Kip1. (C): AsPC-1 cells were transfected with negative control or p27Kip1 siRNA and subjected to the MTT cell proliferation assay (The results are indicated as the absorbance readings at 490 nm). (C) (D, E): AsPC-1 cells were transfected with a negative control mimic, the miR-221 mimic, antisense oligonucleotides to miR-221 (anti-mIR-221) or p27Kip1 siRNA. The cells were then treated with PDGF-BB (20 ng/ml) for 24 h and subjected to staining with a FITC-conjugated antibody against the proliferation marker PCNA and DAPI (E). In total, 150 cells were counted per condition, and the percentage of PCNA-positive cells is presented (D). All experiments in this figure were carried out in triplicate, and the results are displayed as the means ± SD.

p27Kip1 is a member of the Cip/Kip family of cyclin-dependent kinase (CDK) inhibitors that function to negatively regulate cell cycle progression from G1 to S phase by binding to CDK2 and cyclin E complexes [23]. To test the role of p27Kip1 in AsPC-1 cell proliferation, we reduced the endogenous level p27Kip1 by siRNA (si-p27Kip1) (Fig. 5A, B) and measured cell growth over 4 days (Fig. 5C). The proliferation of si-p27Kip1-transfected cells was significantly increased in comparison to control cells, suggesting that decreased expression of p27Kip1 can promote cell growth in AsPC-1 cells. Therefore, we hypothesized that PDGF-dependent promotion of AsPC-1 cell proliferation might be mediated through down-regulation of p27Kip1. Quantitative analysis of PCNA staining showed that p27Kip1 expression was inhibited by PDGF-BB (20 ng/ml) after 24 h of treatment, and transfection of si-p27Kip1 increased the number of proliferating cells to a similar extent as PDGF treatment (Fig. 5 C, D, E). MiR-221 has previously been shown to inhibit p27Kip1 and promote cell proliferation [13], [24], [25]. Therefore, we investigated whether PDGF-mediated proliferation of AsPC-1 cells is affected by miR-221-mediated inhibition of p27Kip1. Transfection of the miR-221 mimic significantly reduced the expression of p27Kip1 (Fig. 5A, B) and elevated cell proliferation (Fig. 5D, E), similarly to cells transfected with si-p27Kip1 (Fig. 5A, B, D, E). When endogenous miR-221 was depleted by anti-miR-221, PDGF-BB treatment did not repress the expression of p27Kip1 (Fig. 5A, B). Thus, the PDGF-dependent increase in cell proliferation appears to be mediated by the inhibition of a specific target of miR-221 and down-regulation of p27Kip1.

## Discussion

A growing body of literature strongly suggests that PDGF may function as a key player in the development and progression of human cancers by regulating the processes of cell proliferation, apoptosis, migration, invasion, angiogenesis, and metastasis. It has been reported that PDGF signaling is frequently deregulated in human malignancies, and upregulated expression of PDGF was found in pancreatic, prostate, lung, renal, ovarian and brain cancers [6]. In recent years, PDGF signaling has been found to play important roles in the acquisition of the EMT phenotype in cancer cells [6], [8], [26]. Moreover, it has become increasingly clear that the EMT plays an important role in the progression of cancer and is also responsible for the resistance of cancer cells to conventional chemotherapeutics. Therefore, PDGF signaling has been considered to be important in human malignancies, and thus, PDGF signaling may represent a novel therapeutic target, suggesting that the development of agents that target PDGF signaling is likely to have a significant therapeutic impact on human cancers. Our current study clearly demonstrated that PDGF-BB treatment promoted cell migration and proliferation and resulted in the acquisition of the EMT phenotype by up-regulation of mesenchymal cell markers (ZEB1, ZEB2, Snail2, and vimentin) and down-regulation of an epithelial cell marker (E-cadherin) in AsPC-1 cells. Thus, targeting PDGF signaling may inhibit the acquisition of the EMT phenotype, which will result in the reversal of drug resistance in the treatment of metastatic disease.

In the current study, we have further demonstrated that miR-221 gene transcription is regulated by PDGF signaling. The PDGF pathway is well known as a modulator of the expression of various protein coding genes, but miR-221 is the first miRNA gene found to be regulated by PDGF. It is intriguing to speculate that PDGF may activate miR-221 transcription through recruitment of microphthalmia-associated transcription factor or other E-box binding proteins. We found that miR-221 was induced 2.5-fold after 4 h of treatment with PDGF, and both the primary transcripts and intermediate products of miR-221 were observed as early as 2 h after PDGF treatment, suggesting that miR-221 expression is activated by PDGF signaling. miR-221 is highly expressed in various cancer-derived cells, including pancreatic carcinoma, prostate carcinoma, and thyroid carcinoma cells [14], [15]. Moreover, miR-221 has been shown to stimulate several aspects of cancer pathogenesis, including metastasis, invasion, and self-renewal [27], [28], [29]. When miR-221 function was blocked by anti-miR-221, we found that PDGF did not alter the level of cell migration and proliferation and result in the acquisition of the EMT phenotype. These results confirm that miR-221 plays an essential role in the PDGF-mediated epithelial-mesenchymal transition phenotype, migration, and growth of AsPC-1 cells. MiR-221 and miR-222 share the same seed sequence, indicating a great degree of functional overlap between these two mRNAs. However, our result indicated that miR-222 does not involved in the PDGF-mediated EMT phenotype and cancer cell migration and proliferation (Figure S1).

A critical role of miR-221 has been demonstrated in several human cell types, including various carcinomas, hematopoietic cells, and endothelial cells [13], [14], [15], [16]. High expression of miR-221 has been associated with increased proliferation of carcinomas or resistance to chemotherapy agents [30], [31], [32]. It was suggested that silencing of the tumor suppressor gene p27Kip1, which leads to proliferation, is a critical role of miR-221 in cancer cells [13], [24], [25], [32]. Our results also confirm that miR-221-dependent down-regulation of p27Kip1 promotes cell proliferation in AsPC-1 cells. p27Kip1 is a member of the Cip/Kip family of cyclin-dependent kinase (CDK) inhibitors that function to negatively control cell cycle progression from G1 to S phase by binding to CDK2 and cyclin E complexes [23]. In addition to its role as an inhibitor of cell proliferation in the nucleus, p27Kip1 is known to interact with RhoA in the cytoplasm to modulate cytoskeletal organization and cell migration [32], [33]. Therefore, we speculate that the miR-221-p27Kip1 axis might also affect the PDGF dependent change in cell migration. However, we did not observe this phenomenon.

This intriguing and emerging evidence demonstrating the regulation of mRNAs by miRNAs in the processes of the EMT prompted us to investigate whether miR-221 could play a role in the PDGF-induced EMT of AsPC-1 cells and to further assess whether the processes of the EMT in our cell culture model system could also be regulated by the ZEB1, ZEB2, and Snail2 transcription factors. We found that miR-221 is a downstream effector of PDGF signaling that promotes the EMT by targeting TRPS1. TRPS1 is a member of the GATA family transcriptional factors, which bind specifically to “GATA” DNA sequences [22]. ZEB2 is positively associated with the EMT in development. ZEB2 directly represses E-cadherin transcription and increases vimentin transcription [9]. Recent studies have shown that ZEB2 could be regulated by TRPS1 based on the presence of GATA sites near its promoter and its increased mRNA abundance in response to miR-221 [16]. Our results confirm that ZEB2 is one of the downstream genes responsible for TRPS1-mediated repression of the EMT. In this study, we demonstrate that PDGF signaling reduces the expression of TRPS1 through up-regulation of miR-221. We observed that exogenous miR-221 significantly reduced the expression of TRPS1, whereas the TRPS1 mRNA level was elevated in the presence of anti-miR-221. More importantly, PDGF treatment did not repress TRPS1 in the presence of anti-miR-221 (Fig. 4B). These results demonstrate that the miR-221 directly targets TRPS1 mRNA, as previously observed [32], [33].

The results of our current study clearly suggest that PDGF exhibits its effects on both cell proliferation and the EMT phenotype by inducing miR-221 expression, which results in down-regulation of p27Kip1 and TRPS1 in pancreatic cancer cells. Our findings raise the possibility that the inhibition of miR-221 function in pancreatic cancer cells, either by treatment with anti-miR-221 or by overexpression of the miR-221 target sequence (miRNA sponge), represents a potential new therapy for proliferative disorders caused by activation of the PDGF pathway.

## Supporting Information

Figure S1 **miR-222 does not involved in the PDGF-mediated EMT phenotype and cancer cell migration and proliferation.** AsPC-1 cells were transfected with a negative control or anti-mIR-221 and subjected to the Matrigel transmembrane invasion assay (A) in the presence of 20 ng/ml PDGF-BB. AsPC-1 cells were transfected with a negative control or anti-mIR-221. The cells were then treated with PDGF-BB (20 ng/ml) for 24 h, and then were stained with a FITC-conjugated antibody against the proliferation marker PCNA and DAPI (presented the percentage of PCNA-positive cells) (B), and subjected to qRT-PCR (C, D,E) of the transcription factors and EMT-specific gene markers. All treatment experiments in this figure were carried out in triplicate, and the results are displayed as the means ± SD.
(DOC)
