# Supplementary material for: MicroRNA-221 Mediates the Effects of PDGF-BB on Migration, Proliferation, and the Epithelial-Mesenchymal Transition in Pancreatic Cancer Cells
Source: PLoS One. 2013 Aug 13;8(8):e71309. doi: 10.1371/journal.pone.0071309 (PMC3742757; doi:10.1371/journal.pone.0071309)
Supplement: Figure S1 — miR-222 does not involved in the PDGF-mediated EMT phenotype and cancer cell migration and proliferation. AsPC-1 cells were transfected with a negative control or anti-mIR-221 and subjected to the Matrigel transmembrane invasion assay (A) in the presence of 20 ng/ml PDGF-BB. AsPC-1 cells were transfected with a negative control or anti-mIR-221. The cells were then treated with PDGF-BB (20 ng/ml) for 24 h, and then were stained with a FITC-conjugated antibody against the proliferation marker PCNA and DAPI (presented the percentage of PCNA-positive cells) (B), and subjected to qRT-PCR (C, D,E) of the transcription factors and EMT-specific gene markers. All treatment experiments in this figure were carried out in triplicate, and the results are displayed as the means ± SD. (DOC) [file pone.0071309.s001.doc]

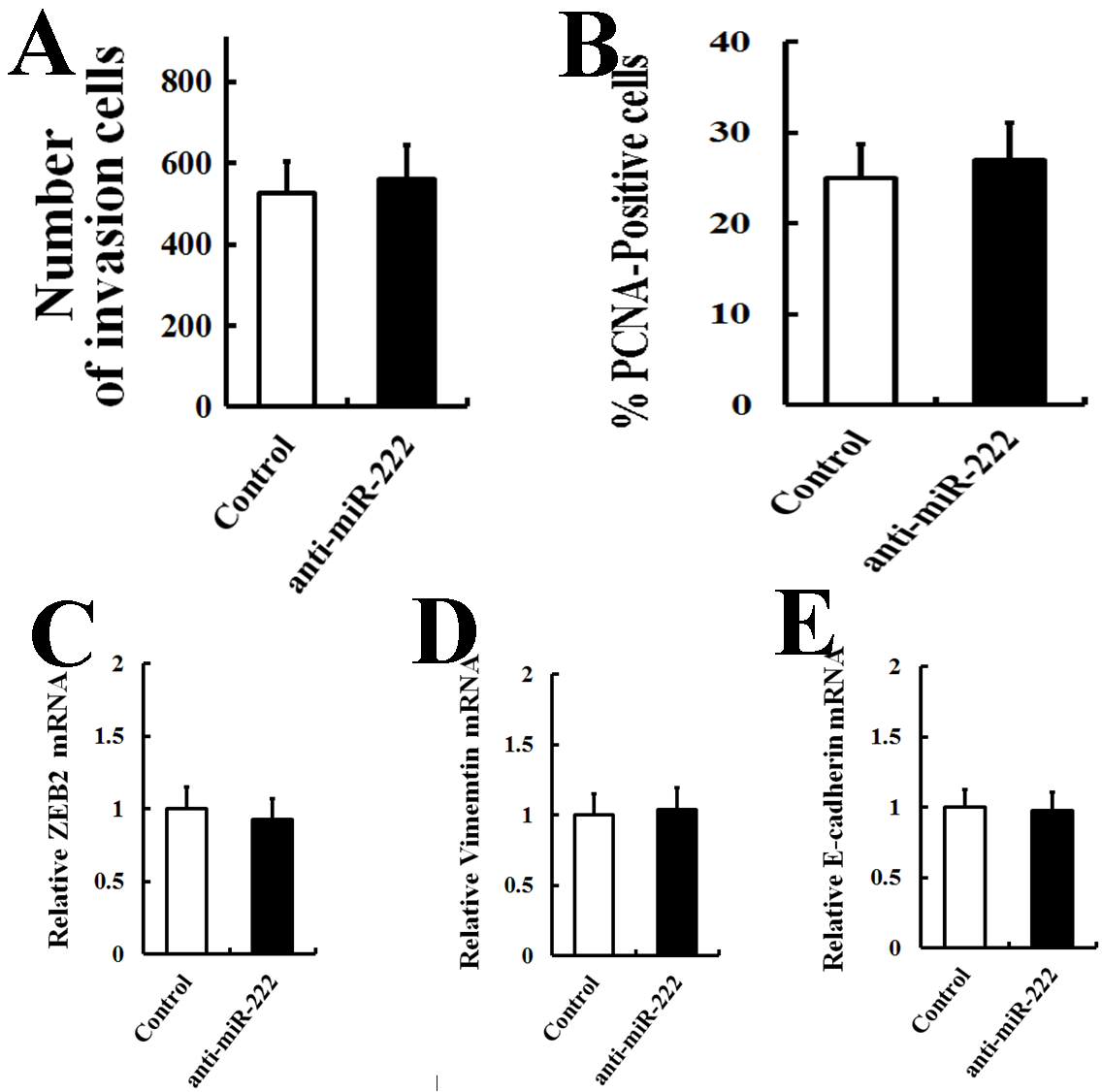


**Figure S1: miR-222 does not involved in the PDGF-mediated EMT phenotype and cancer cell migration and proliferation**

AsPC-1 cells were transfected with a negative control or anti-mIR-221 and subjected to the Matrigel transmembrane invasion assay (A) in the presence of 20 ng/ml PDGF-BB.

AsPC-1 cells were transfected with a negative control or anti-mIR-221. The cells were then treated with PDGF-BB (20 ng/ml) for 24 h, and then were stained with a FITC-conjugated antibody against the proliferation marker PCNA and DAPI (presented the percentage of PCNA-positive cells) (B), and subjected to qRT-PCR (C, D ,E) of the transcription factors and EMT-specific gene markers.

All treatment experiments in this figure were carried out in triplicate, and the results are displayed as the means ± SD.
